# *In*-*situ* Electrodeposition of Highly Active Silver Catalyst on Carbon Fiber Papers as Binder Free Cathodes for Aluminum-air Battery

**DOI:** 10.1038/s41598-017-03609-9

**Published:** 2017-06-13

**Authors:** Qingshui Hong, Huimin Lu

**Affiliations:** School of Materials Science and Engineering, Beihang University, Beijing, 100191 China

## Abstract

Carbon fiber papers supported Ag catalysts (Ag/CFP) with different coverage of electro-active site are prepared by electrochemical deposition and used as binder free cathodes in primary aluminum-air (Al-air) battery. Scanning Electron Microscopy and X-ray Diffraction studies are carried out to characterize the as-prepared Ag/CFP air cathodes. Oxygen reduction reaction (ORR) activities on these air cathodes in alkaline solutions are systematic studied. A newly designed aluminum-air cell is used to further determine the cathodes performance under real operation condition and during the test, the Ag/CFP electrodes show outstanding catalytic activity for ORR in concentrated alkaline electrolyte, and no obvious activity degradation is observed after long-time discharge. The electrochemical test results display the dependence of coverage of the electro-active Ag on the catalytic performance of the air cathodes. The resulting primary Al-air battery made from the best-performing cathode shows an impressive discharge peak power density, outperforming that of using commercial nano-manganese catalyst air electrodes.

## Introduction

Alkaline metal-air batteries have been proposed as indisputable promising candidates for safe, green and large-scale electricity storage technology due to their advantages of using non-platinum electrode catalysts and less corrosive electrolyte than acidic media, possessing extremely high energy density and no emission of greenhouse gases^1–5^. For metal-air batteries in alkaline media, the active metal, including lithium, magnesium, aluminum, iron and zinc, is oxidized at the anode, releasing electrons that travel from the external circuit to the air cathode. Meanwhile, the oxygen molecule receives the electrons and then is reduced to hydroxide ions at the cathode, namely, oxygen reduction reaction (ORR)^6–11^. As being the most abundant metal in earth’s crust and possessing very low molecular mass, Al is an attractive candidate for alkaline metal-air batteries. Indeed, the Al-air battery possesses high theoretical specific energy capacity (2980 A h kg^−1^), which is the second highest after that of lithium (3860 A h kg^−1^), and is capable of outputting high current densities^12–14^. However, it is still a challenge to successful large-scale commercialize Al-air battery because of its sluggish kinetics of the cathodic process, which increases overpotential dramatically in the discharge step and leads to battery performance degradation. The key approach to implement this technology is to create a stable and highly efficient air electrode with low over-potential under high current density^15^. Developing highly efficient, low cost and durable electrocatalyst for ORR at the air electrode is a critical technology for fabrication of these air electrodes^16–20^. The generally used precious Pt and its corresponding alloys are widely regarded as the most efficient catalyst for the ORR, however, they suffer from high cost, limited stability and fast deactivation^21–25^. Therefore, a much wider range of less expensive metal catalysts that are stable in alkaline environments are explored to replace the Pt-based catalyst in development of air electrode, including nickel, cobalt, silver, copper and manganese^26–30^. For instance, the QuantumSphere incorporated company has developed a high-performance air breathing gas diffusion electrode by using nano manganese catalyst for commercial use in metal-air batteries. Among the studied metal catalyst, Ag-based catalysts are the most promising materials to replace Pt due to its reasonably high activity, good long-term stability (as the equilibrium potential of Ag/Ag_2_O being ~200 mV higher than that of Pt/PtO) and relatively low price (~2% the price of Pt). Although several research groups have proved the ORR activity of Ag electrocatalysts in alkaline media by density functional theory calculations and experiments, there are few reports about preparing and evaluating Ag-containing air electrodes that can operate in real air batteries^27, 31–34^.

For commercial use, it has been generally expected that the air electrodes must not only minimize the catalysts loading but must also improve the catalytic performance as well as stability under realistic operation conditions. A desired support material with proper structure may overcome these barriers^35^. And more importantly, the architecture of the support substrate should be highly porous that can facilitate the diffusion of oxygen and electrolyte to reach the active sites within the air cathode^36^. Recently, the three-dimensional (3D) carbon fiber material (e.g. CFP and carbon felt), a well-suited substrate for supporting electrocatalyst with a 3D porous network, high electron conductivity and robust mechanical stability, has been extensively utilized as an electrode substrate to fabricate electrochemical devices^37–40^. Compared with 3D carbon felt, more progress has been made to develop CFP-based air electrode due to its highly porous and low density, which can provide an unobstructed gas-diffusion pathway to continually supply enough oxygen reactant to the reactive catalytic sites in the cathode^41, 42^. In previous reports, CFPs integrating with electrocatalytic functional materials have shown high ORR performance. The merging of conductive carbon fiber with active catalyst results in robust 3D networks that favor the mass transfer from the electrolyte to the electrode and simultaneously the electron transfer to the active sites and then to the adsorbed oxygen molecules^40, 43–45^. However, synthesis most of these air electrodes requires rigorous techniques, such as high vacuum conditions^43^, complex and expensive multiple steps^8, 44^, using excessive, expensive and toxic organic ligands to control morphology^43^, and utilizing persistent polymer binders^46–48^, hampering their widespread commercial application.

Thus, it would be an effective strategy to couple Ag-based catalyst with CFP substrate for much improving the performance of air electrode. Better still, it is necessary to develop easy, green, and one step *in*-*situ* mild synthesis methods for fabrication binder free air electrodes. In this work, we developed a low-cost and facile *in*-*situ* synthesis process to construct highly integrated Ag catalyst layer and CFP without utilizing hazardous polymer binder for the first time. The Ag precursor was self-assembled onto the functionalized CFP surface (pretreated with concentrated acid) through electrostatic interaction between Ag^+^ and negatively charged functional groups followed by being slowly reduced and formed metallic Ag particles. The newly developed binder free Ag/CFP air electrodes display excellent and stable electrocatalytic performance towards the ORR, along with exceptionally high performance for primary Al-air battery. In addition, the electrochemical analysis represents that the particle size and dispersion of Ag catalyst on the substrate surface are critical for the catalytic activity and display the dependence of coverage of the electro-active Ag on catalytic performance.

## Results

### Preparation of binder free Ag/CFP electrodes

**Figure 1:**
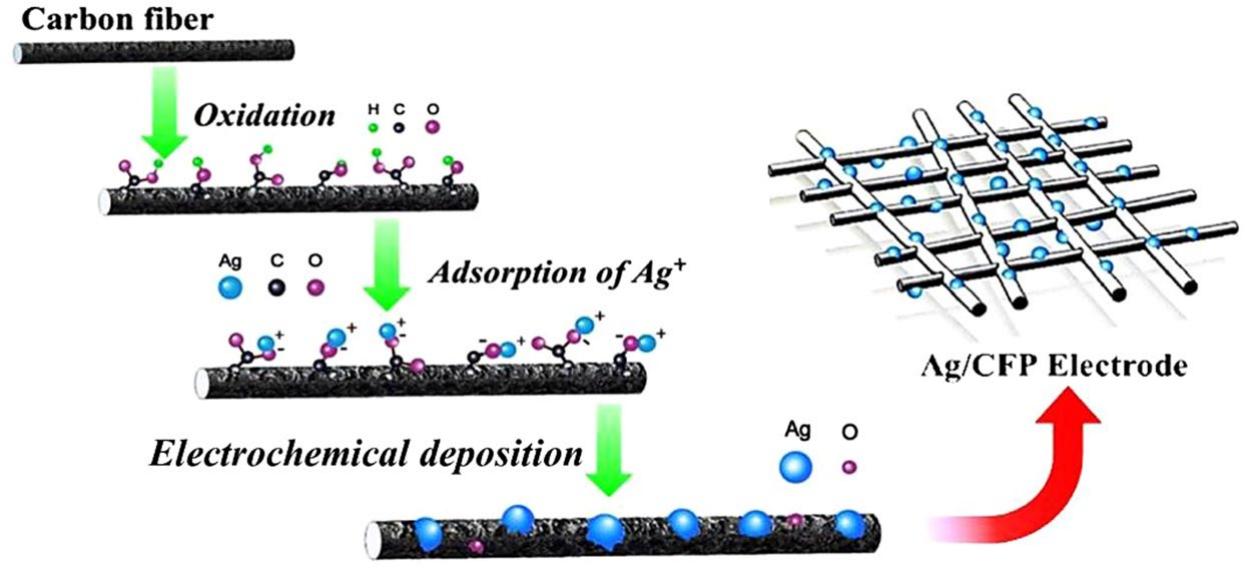
Schematic diagram of the synthesis process of Ag/CFP electrode.

The fabrication of Ag/CFP electrode is shown in Fig. 1. The commercial CFP substrate was first mildly oxidized by concentrated H_2_SO_4_/HNO_3_ to create abundant negatively charged functional groups (e.g. –COO^−^) on the surface. The resulting surface oxidized CFP was immersed in an aqueous solution containing 2 mM Ag precursor. With the surface functionalization, the CFP and Ag precursor were self-assembled through electrostatic interaction, and then the bound Ag^+^ was localized to *in*-*situ* nucleate/grow metallic Ag (see Figure S2, Supporting Information)^49^. Though careful selections of electrodeposition potential, we directly obtained binder free Ag/CFP air electrodes with controlled Ag particle size and distribution. The composition and morphology of the Ag/CFPs were characterized and analysed in detail.

### Material Characterization

**Figure 2:**
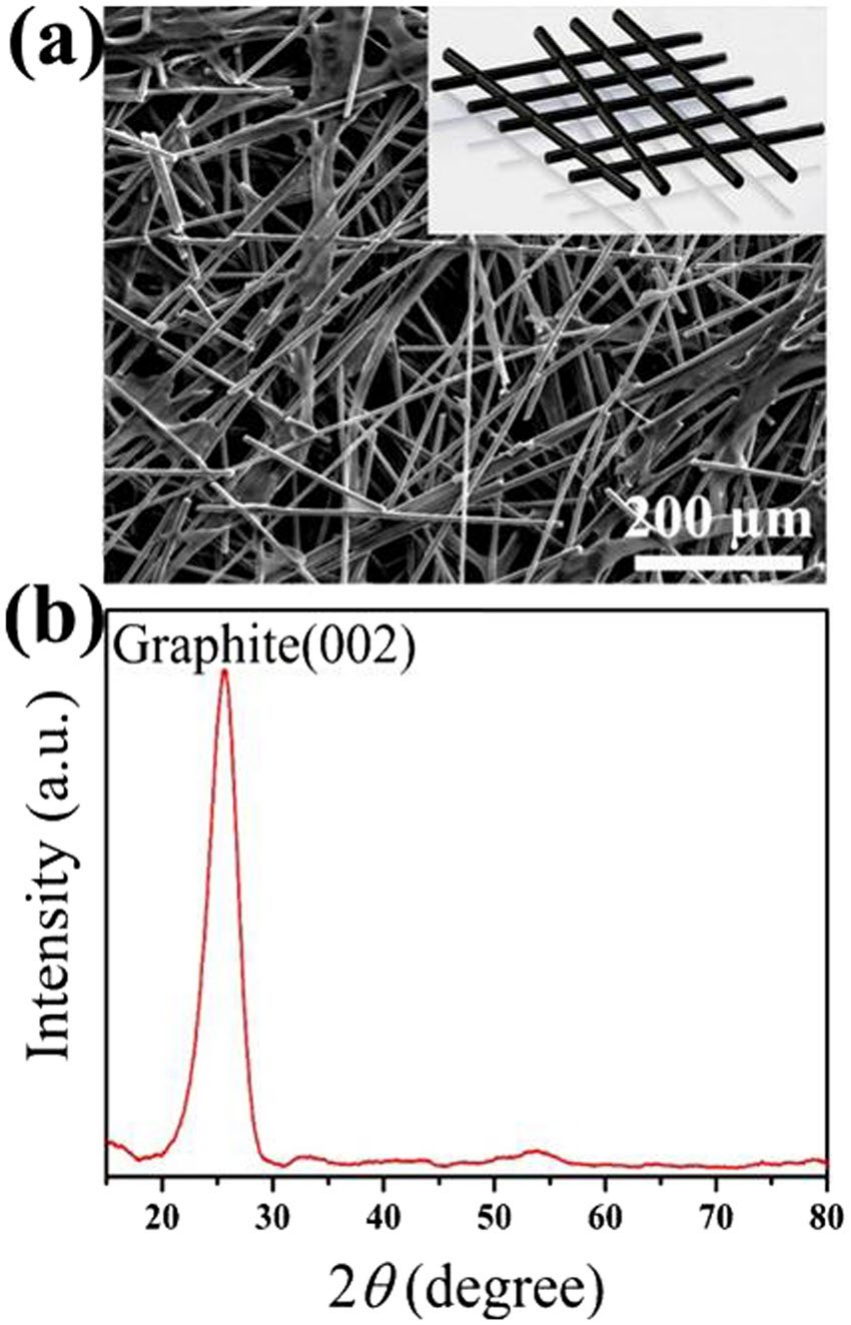
(**a**) SEM image and (**b**) XRD spectra of the pristine CFP. Inset in panel (a): Schematic illustrating the interpenetrating 3D network structure of CFP.

In practical application, porous current collectors, such as foams of metal and carbon fiber frameworks, have been extensively employed as electrode substrates to fabricate electrochemical devices^42^. Due to its good electrochemical stability and easy modification, CFP is a well-suited substrate for supporting electrocatalysts. As shown in Fig. 2a, CFP has a 3D network consisting of well-connected carbon fibers with a diameter of ~10 µm. Figure 2b shows the typical XRD pattern of the bare CFP. The intensive peak centered at 25.5° is attributed to the graphite (002) plane of CFP substrate. This result suggests that a presence of hexagonal structure carbon atoms in the carbon fiber may enable fast electron transport to all fiber networks^50, 51^.

**Figure 3:**
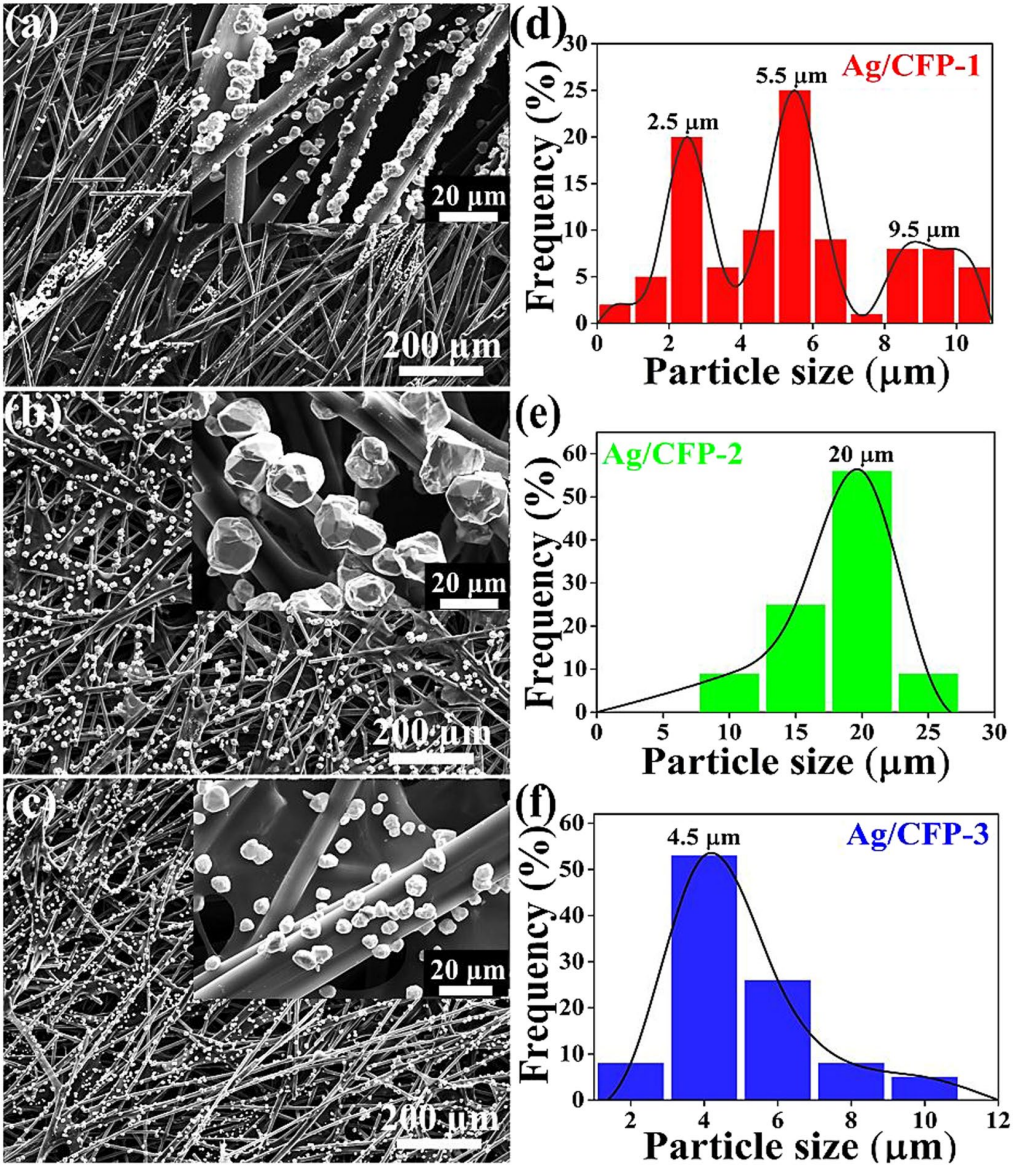
SEM images and magnified surface images (inset) of (**a**) Ag/CFP-1, (**b**) Ag/CFP-2 and (**c**) Ag/CFP-3 and (**d**–**f**) their corresponding histograms of size distribution statistics of Ag particles on the surface of CFPs.

To probe the effect of particle size and distribution of Ag electrocatalyst on the catalytic performance, we prepared batches of Ag/CFP electrodes by controlling the deposition potential progressively more negative while the serving time remained constant. Figure 3a–c shows representative SEM images of Ag electrodeposited on the CFPs under the applied voltages of 1.5, 3 and 4.5 V, respectively. It can be clearly seen that the Ag particles coated firmly on the carbon fibers exposed in the CFP for all the samples. Note that the adhesion between Ag particles and carbon fiber surface is sufficient to survive the washing and drying process. The corresponding magnified SEM images (inset of Fig. 3) show that the coating of Ag particles on carbon fibers of the three samples possesses different particle size distribution. The size distribution of Ag particles in individual electrode is summarized in the Fig. 3d–f, where over 100 samples were counted. In details, the mean size of Ag particles in Ag/CFP-1, Ag/CFP-2 and Ag/CFP-3 are 5.4, 20 and 4.5 µm, respectively, which increases firstly and then decreases with the applied nucleation voltage progressively more negative. It is note that the nucleation potential *E*_*n*_ is critical for formation of Ag particle and thus the particle size and surface coverage can be controlled by the applied potential^52^. Small particles and part of continuous layers are apparent on the fiber side walls at low coverage for *E*_*n*_ = −1.5 V (see Fig. 3a). The size of the particles and coverage increase significantly as the potential is made more negative and at *E*_*n*_ = −3 V, the carbon fiber sidewall is uniformly covered by Ag particles with a relatively narrow particle size distribution (see Fig. 3b). Nevertheless, the particle size deposited decrease under conditions where instantaneous nucleation and diffusion-controlled growth dominate (sufficiently negative potential) and at *E*_*n*_ = −4.5 V, we do indeed observe relatively smaller particles on the carbon fiber side wall (see Fig. 3c).

**Figure 4:**
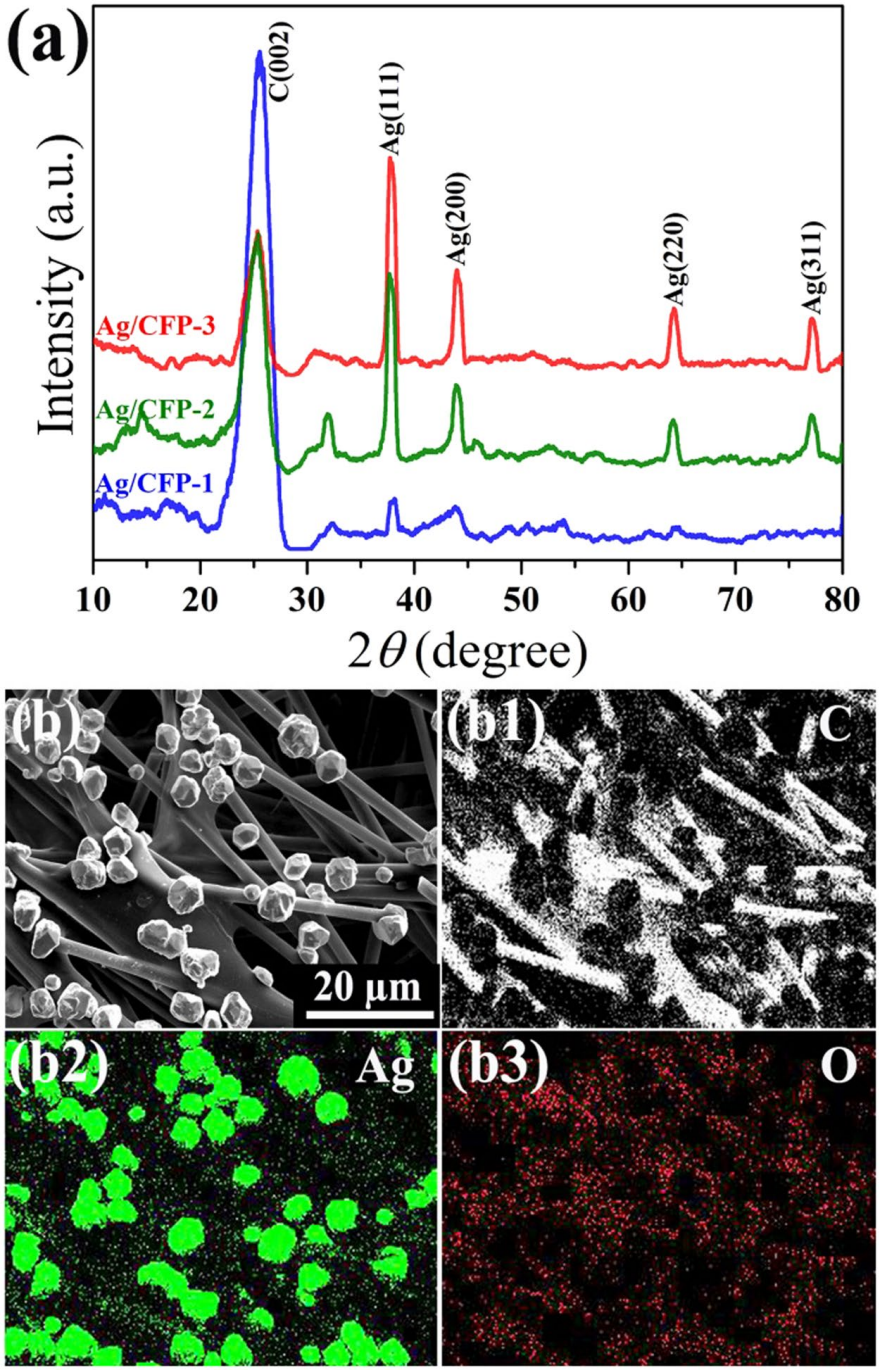
(**a**) XRD patterns of Ag/CFP-1, Ag/CFP-2 and Ag/CFP-3. (**b**) SEM image of Ag/CFP-3 used for elemental mapping analysis, (b1) C map, (b2) Ag map and (b3) O map.

The composition and structure of the three electrodes were further characterized by XRD. As shown in Fig. 4a, the diffraction peaks at 38.1° (111), 44.3° (200), 64.4° (220) and 77.5° (311) indicate the formation Ag with face-centered-cubic structure (JCPDS card No. 4-783). It is observed that the Ag diffraction peaks are intensified by increasing surface coverage of particles. There are no obvious characteristic peaks of Ag oxide detected, indicating the absence of oxide layer formation during the synthesis. The intensive peak at 25.5° referring to the graphite (002) planes from carbon fiber was also observed in the XRD pattern. The typical elemental mapping analysis of Ag/CFP (selection of Ag/CFP-3 as representative samples) was conducted by EDS, which confirms the presence of Ag, C and O elements in the sample (Fig. 4b). It is proposed that the facile and green synthetic strategy, by coupling *in*-*situ* depositon with self-assembly, enable the fabrication of metallic particles onto complex surface with good crystallinity and dispersibility.

### Activity of Ag/CFP electrodes for ORR

**Figure 5:**
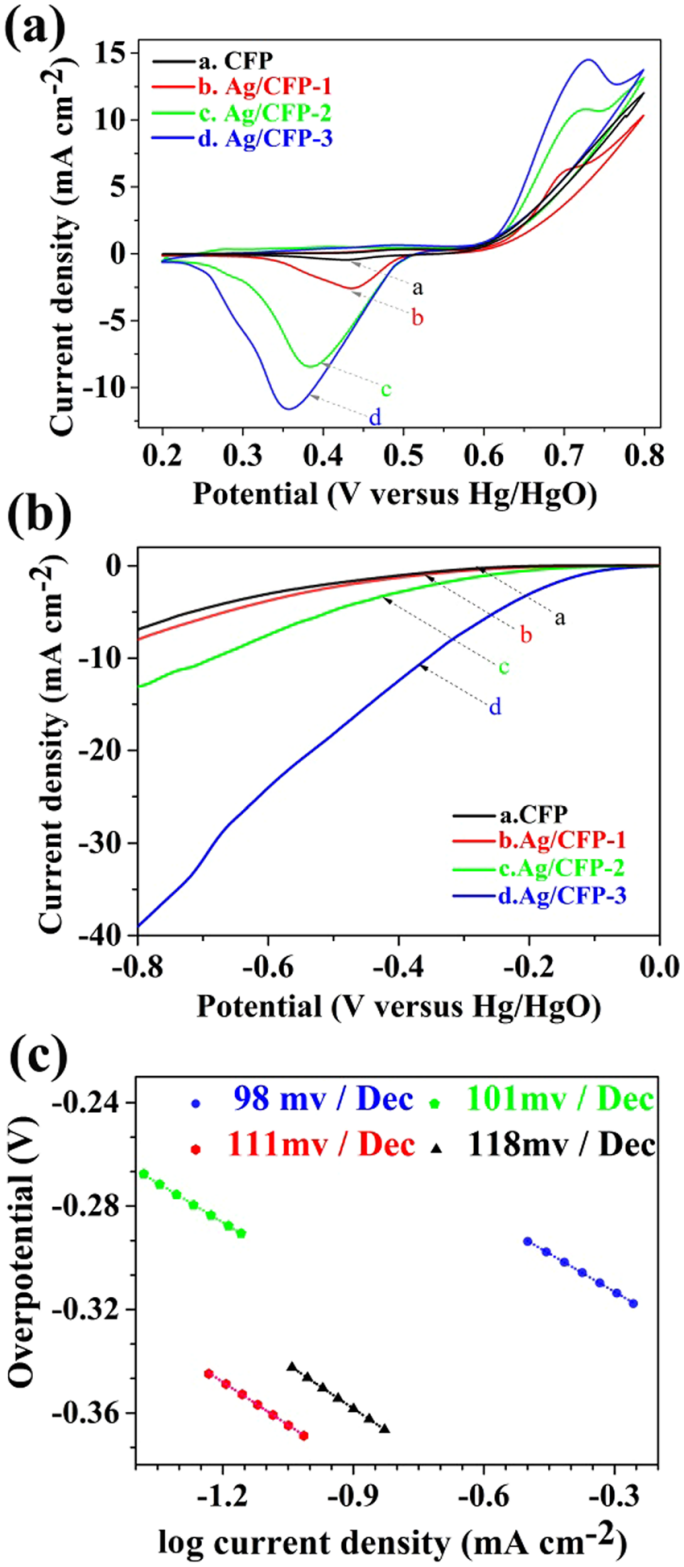
(**a**) Cyclic voltammograms, (**b**) linear sweep voltammograms and (**c**) Tafel plots of CFP (black line), Ag/CFP-1 (red line), Ag/CFP-2 (green line) and Ag/CFP-3 (blue line), in O_2_-purged 0.1 M NaOH solution.

Ag/CFP can be directly used as the working electrode for ORR without extra substrates or binders. We investigated the electrocatalytic ORR performance of the four samples in an oxygen purged 0.1 M NaOH solution using a standard three-electrode system. Before start of CV data collection, the electrodes were cycled for several times until the signals were stabilized. Figure 5a compares the CV curves of CFP and Ag/CFP electrodes prepared by electrochemical deposition. In the potential range of 0.6–0.8 V vs Hg/HgO, there is a distinct oxidative peak observed on the Ag/CFP electrode, yet not seen for the CFP, which is associated with the formation of a surface monolayer of Ag_2_O film^53^. In the forward cathodic potential scan, there are obvious reductive peaks commenced at potential of around 0.53 V vs Hg/HgO on the three Ag/CFP electrodes. The availability of electro-active Ag sites can be calculated by integrating the charge across the reductive waves (from onset potential of 0.53 V to the ending of 0.2 V) using the following equations:

1 $${C}_{Ag}={\int }_{0}^{t}\,jdt$$

2 $${{\Gamma }}_{Ag}=\frac{{C}_{Ag}{M}_{Ag}}{F}$$

3 $$A{g}_{act} \% =\frac{{{\Gamma }}_{Ag}}{{W}_{Ag}}\cdot 100 \% $$

where *C*_*Ag*_ (mC cm^−2^) is the accumulative charge obtained by integration of the reductive waves, *j* (mA cm^−2^) is the current density, *t* is the time at the scan potential of 0.2 V, *Γ*_*Ag*_ (mg cm^−2^) is the coverage of the electro-active Ag, *M*_*Ag*_ is atomic weight of Ag, *W*_*Ag*_ (mg cm^−2^) is the total Ag loading on the electrode, *F* is Faradic constant, and *Ag*_*act*_ *%* is the percentage of electro-active Ag. The calculation results show highly consistent with SEM analysis, which are summarized in Table 1. In the case of samples by electrodeposition, the *Γ*_*Ag*_ and *Ag*_*act*_% are shown to go up as electrodeposition potential increased. The Ag/CFP-3 has the maximum value of *Ag*_*act*_%, up to 9.561%, approached the best reported (ca. 10%), to our knowledge^54^. This considerable percentage of electro-acitve Ag site arising from the small particle size and high specific surface area can guarantee both a minimization of the catalyst loading and a high catalytic performance of the electrode.

**Table 1:** Comparison of the electrode activity of the as-prepared Ag/CFPs.

Electrode material	Catalyst loading (mg cm^−2^)	*Γ*_*Ag*_ (mg cm^−2^)	*Ag*_*act*_ *%*	ORR onset potential (V vs Hg/HgO)	ORR tafel slope (mV dec^−1^)
Ag/CFP-1	1.02	0.0278	2.725	−0.29	111
Ag/CFP-2	3.56	0.1294	3.634	−0.22	101
Ag/CFP-3	1.89	0.1807	9.561	−0.07	98

The linear sweep voltammograms and corresponding Tafel plots for ORR activity of the samples are shown in Fig. 5b and c, respectively. The pristine CFP exhibits negligible activity, while the Ag/CFP-3 affords an ORR onset potential of −0.07 V (vs. Hg/HgO) more positive to that of Ag/CFP-1 (−0.29 V) and Ag/CFP-2 (−0.22 V). The parameters of ORR activity of each sample are summarized in Table 1. An enhancement of performance on Ag/CFP-3 is indicated by its smaller Tafel slope (98 mV dec^−1^). The more positive onset potential indicates a more facile ORR process initiated on the electrodes^43^. Thus, the best-performing cathode during the LSV tests is Ag/CFP-3. Furthermore, the results of *Γ*_*Ag*_ and ORR activity of each sample were summarized in Table 1 for comparison. It is noted that the catalytic performance of the electrode shows dependence on the coverage of the electro-active Ag on the samples. This comparison indicates that the higher coverage of the electro-active Ag results in faster catalytic ORR rate towards the air cathodes with the same size. Consequently, the high ORR catalytic activity of Ag/CFP-3 suggests that the particle size and dispersion of Ag catalyst on the CFPs surface are critical for improving the ORR activity.

**Figure 6:**
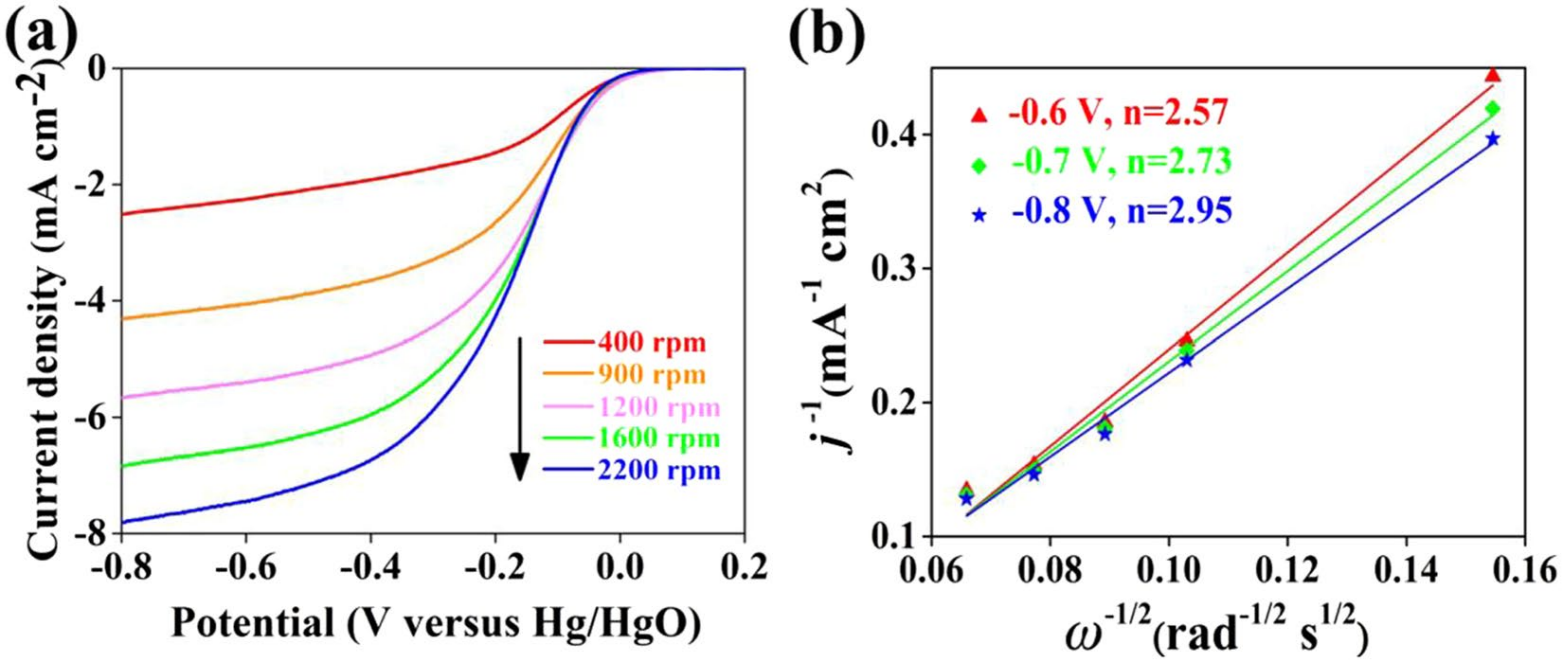
(**a**) ORR polarization curves for Ag/CFP-3 at different rotating rates. (**b**) Koutecky-Levich plots collected from the ORR.

The electrochemical reduction of O_2_ in alkaline solution is a multi-step electron transfer reaction that has two main possible pathways: one involving the transfer of two electrons to produce OH_2_^−^ and the other involving a direct four-electron pathway to produce OH^−^^24^. We also performed RDE test with the ground catalyst from the best performance electrode to determine the electron transfer number *n* during a typical ORR process on the Ag/CFP air cathode. The measured current densities at different rotation speeds and different potentials from −0.6 V to −0.8 V, in which the inaccuracy of the mass transport correction is relatively small^21^, are used to construct the Koutechy-Levich (K-L) plots as shown in Fig. 6. The overall *n* per oxygen molecule can be calculated from the slopes of K-L plots using the following equation:

4 $$1/j=1/{j}_{k}+1/B{{\omega }}^{1/2}$$

5 $${j}_{k}=nFk{C}_{{0}}$$

where *j* is the measured current density, *j*_*k*_ is the kinetic current in amperes at a constant potential, *ϖ* is the angular velocity of the disk in the radian, *k* is the electron transfer rate constant and *B* is the reciprocal of the slope determined from K-L plots based on the Levich equation:

6 $$B=0.62nF{{D}_{{0}}}^{2/3}{v}^{-1/6}{C}_{{0}}$$

where *n* is the total number of electrons transferred during the ORR test, *F* is the Faraday constant (96 485 C mol^−1^), *D*_*0*_ is the diffusion coefficient of O_2_ in 0.1 M NaOH (1.9 × 10^−5^ cm^2^ s^−1^), *v* is the kinematic viscosity of the electrolyte (0.01 cm^2^ s^−1^), *C*_*0*_ is saturation concentration of O_2_ in 0.1 M NaOH at 1 atm O_2_ pressure (1.2 × 10^−6^ mol cm^−3^). As shown in Fig. 6a, the RDE data of Ag/CFP-3 show that the current density increases with increasing rotating rate, indicating that the limiting current density is controlled by the diffusion distance of O_2_ to the Ag catalyst surface. Figure 6b shows its corresponding K-L plots at different potentials, which display relatively good linearity with a similar slope, suggesting first-order reaction kinetics toward the concentration of dissolved oxygen. The *n* per oxygen molecule was analyzed according to the K-L equations. Derived from the plot slopes, the average number of electrons transferred on Ag/CFP-3 is calculated to be 2.75. The result suggests that the ORR catalyzed by the Ag/CFP simultaneously occurs through four-electron pathway and two-electron pathway. According to the previous researches, it is very likely that the number of the electrons transferred for Ag particles is closed to 4, but the fact of less than four electrons transferred on Ag/CFP is probably arisen from the ORR catalyzed by the surface of exposed CF through a two-electron pathway^53^.

### Primary Al-air batteries test

**Figure 7:**
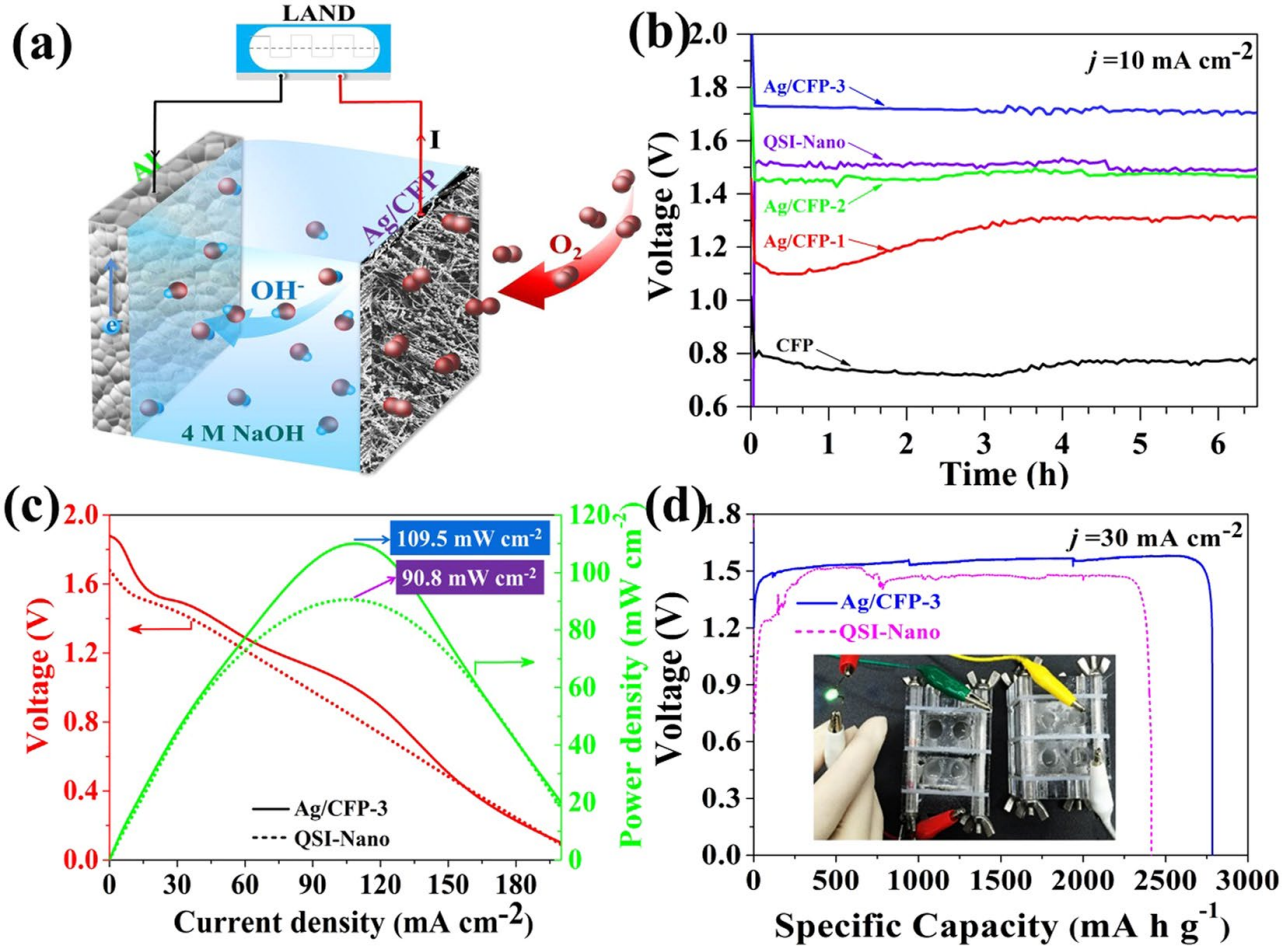
(**a**) A schematic of the primary Al-air battery. (**b**) Long-time discharge curves of primary batteries using bare CFP, Ag/CFP-1, Ag/CFP-2 and Ag/CFP-3 cathodes at current density of 10 mA cm^−2^ compared with the battery using commercial QSI-Nano air electrode as cathode. (**c**) Polarization curve (red) and corresponding power density plot (green) of the batters using Ag/CFP-3 (solid line) and QSI-Nano electrode (dotted line) as the cathodes. (**d**) Specific capacities of the primary Al-air batteries with Ag/CFP-3 and QSI-Nano electrode as the cathodes at the current density of 30 mA cm^−2^, which were normalized to the mass of the consumed Al. The inset shows a photograph of a green LED (≈3.0 V) powered by two liquid Al-air batteries with the Ag/CFP-3 air cathode connected in series.

To further determine the performance of the as-prepared electrodes under real battery operation conditions, we directly used the Ag/CFP electrodes as the air cathodes to construct primary Al-air batteries (Fig. 7a). The cathode was separated by a nylon polymer membrane with the Al metal anode. The use of hydrophobic CFP as the current collector was well-suited to provide a highly developed three-phase boundary for the ORR. Notably, 4 M NaOH was utilized in the battery because of its more negative corrosion potential than concentrated KOH solutions for Al alloy^55^. The galvanostatic discharge curves in Fig. 7b clearly reveal that the performance of Ag/CFP-based Al-air batteries are significantly improved with the increasing of *Γ*_*Ag*_, and finally superior to the commercial QSI-Nano-based battery. The relatively steady curves of long-time galvanostatic discharge indicate a good catalytic stability of Ag/CFP cathodes for the ORR. Although the sliver particles are unstable for the dissolution in concentrated alkali at open circuit, the air cathode potential drop to less than 0.0 V when the ORR occurs, as a result of stabilizing Ag in a form of metallic state^53^. The discharge polarization curves shown in Fig. 7c demonstrate the better performance of Ag/CFP cathodes than the commercial QSI-Nano-based cathode. The peak power densities of Ag/CFP-3 and QSI-Nano-based batteries are 109.5 mW cm^−2^ at 0.98 V and 90.8 mW cm^−2^ at 0.81 V. Both the discharge voltage at 10 mA cm^−2^ and peak power density of Ag/CFP with less mass loading on the electrode are outperformed the battery made with QSI-Nano-based cathode, which are attributed to the strong coupling effect between exceeding ORR activity of the as-prepared Ag particles and the conducting CFP substrate. As discharge continued, the Al plate was gradually thinned, and the electrolyte accumulated more and more soluble meta-aluminate. The battery eventually ceased functioning as the Al plate depleted (Fig. 7d). Furthermore, we tested the battery property at a higher current density of 30 mA cm^−2^ and finally depleted the Al plate. The specific capacity normalized to the mass of consumed Al is shown in Table 2. The best-performing cathode is Ag/CFP-3 with the maximum specific capacity density of 2783.5 mA h g^−1^ and energy density of 4342.3 W h kg^−1^. Note that replenishing the Al plate and electrolyte could reestablish the battery for subsequent runs at the same performance level, again suggesting the durability of Ag/CFP electrode(see Figure S1, Supporting Information).

**Table 2:** A detailed comparison of the Al-air batteries using the as-prepared Ag/CFP-3 electrode and the commercial QSI-nano Mn electrode with key performance parameters.

Cathode material	Catalyst loading (mg cm^−2^)	*E*_*cell*_ @ *j* = 30 mA cm^−2^ (V)	Peak power density (mW cm^−2^)	Specific capacity density (mA h g^−1^)	Energy density (W h kg^−1^)
Ag/CFP-3	1.89	1.56	109.5	2783.5	4342.3
QSI-nano Mn	45	1.47	90.8	2411.8	3545.3

It is evident that the performance (both battery discharge current density and peak power density) of as-prepared Ag/CFP electrode is superior to the commercial QSI-nano air electrode. Moreover, an eloquent comparison of the Al-air batteries property with different cathodes, currently being heavily investigated, is made in Table 3. Only data collected in alkaline electrolytes and at atmospheric air without oxygen feed were taken into account for the sake of proper comparison between systems. Both the discharge current density at 1.5 V and the peak power density are significantly improved over previous reports on Al-air batteries, by at least a factor of 2–20 in current density and 3–15 in power density. These high performances of Al-air batteries using Ag/CFP electrodes are attributed to the strong coupling effect between well dispersive Ag catalyst and 3D conducting CFP substrate. Thus, our Ag/CFP electrode shows significant activity and durability towards the ORR during the battery discharge, and that offers an opportunity to use the electrode for applying in the refueling primary Al-air battery.

**Table 3:** A survey of the Al-air batteries in alkaline electrolytes and at atmospheric air without oxygen feed with key parameters extracted from the literature.

Cathode material	Electrolyte	Current density @ *V* = 1.5 V (mA cm^−2^)	Peak power density (mW cm^−2^)	Reference
Ag/CFP	4 M NaOH	40	109.5	This work
LiMn_2_O_4_/N-rGO/CFP	6 M KOH	≪10	12	56
N-rGO/CFP	6 M KOH	≪10	7	57
60% Pt/C	3 M KOH/CH_3_OH + Alkali anion exchange membrane + 3 M KOH/H_2_O	2	28	58
Ag powder-Nickel mesh	4 M NaOH	10	40	59, 60
Co_3_O_4_/N-doped Ketjenblack/Nickel foam	6 M KOH + Na_2_SnO_3_, In(OH)_3_ and ZnO	20	30	61

## Discussion

In summary, high-performance Ag catalyst coated on the CFPs were synthesized and evaluated for ORR in alkaline electrolyte for the first time. The electro-active Ag dispersed on the surface of CFP is expected to promote the ORR rate by improving the interactions between O_2_ molecules and catalyst surface. Our study shows that the higher coverage of the electro-active Ag results in faster catalytic ORR rate on the entire composited electrodes. We construct primary Al-air batteries using the Ag/CFP electrodes as the air cathode. The best performance battery delivers large discharge current densities (>100 mA cm^−2^) with an excellent peak power density of 109.5 mW cm^−2^, high specific capacity density of 2783.5 mA h g^−1^ and energy density of 4342.3 W h kg^−1^.The discharge tests show that the Ag/CFP air electrode exhibits higher ORR activity than the commercial air electrode under realistic battery conditions. These remarkable performance merits are attributed to the advantages of the 3D skeleton of the CFP substrate, the well dispersive Ag catalyst with superior ORR activity and fast electron transport between current collector and catalytic active site. Thus, we succeed in designing advanced binder free air cathodes with mono-metallic catalyst for high power density primary Al-air battery. And this Ag/CFP air electrode, with its features of easy and green fabrication, high electrocatalytic activity and good stability, provides a promising alternative to replace noble metal Pt based air cathodes in alkaline media, which is suitable for large scale practical application in the low-cost and high performance alkaline fuel cells and metal-air battery.

## Methods

### Preparation of Ag/CFP electrodes

Commercially available CFP was purchased from CeTech Co. Ltd. (340 µm thick, <10 mΩ∙cm^2^, GDL340) to serve as porous current collector. Firstly, the CFP was firstly treated with H_2_SO_4_/HNO_3_ (3:1, v/v) solution at 80 °C for 5 h. Then the pre-treated CFP was rinsed with distilled water and oven-dried at 80 °C for 1 h. As a result of pre-treatment, there was an increase in the amount of surface oxygen-containing functional groups (e.g. −COO^−^, −OH) on CFP, which were able to create active sites to absorb Ag cation.

As illustrated schematically in Fig. 1, samples of Ag/CFP electrodes were synthesized by *in*-*situ* electrochemical deposition. The typical deposition processes were as follows: a simple circuit powered by a DC stabilized power supply (QW-MS3010D, BST Tec. Co., Ltd.) was used to electroplate Ag onto the surface of CFPs with a Pt foil (2 × 2 cm^2^) attached to the positive electrode and the pre-treated CFP (1 × 1 cm^2^) attached to the negative. And then, the two electrodes were immersed in a pre-made AgNO_3_ (2 mM) solution immediately. The electrolytic deposition process was conducted under a range of potentiostatic voltages for 50 s^62^. The value of deposition voltage was set to 1.5, 3 and 4.5 V, respectively, namely Ag/CFP-1, Ag/CFP-2 and Ag/CFP-3. All the resulting samples above were washed with distilled water and alcohol 3 times to remove any extra ions and blown dry with nitrogen. The mass loading of Ag on CFP was determined by the microbalance.

### Material Characterization

The morphologies and compositions of the CFPs before and after deposition of Ag were characterized using a CamScan-3400 scanning electron microscopy (SEM) coupled with energy dispersive X-ray spectroscopy (EDS). The crystal structures of the samples were evaluated using a Rigaku D/MAX 2000 X-ray generator and diffractmeter with Cu-Kα radiation (0.154056 nm).

### Electrochemical Measurement

Electrocatalytic activity of individual Ag/CFP electrode was measured in a homemade three-electrode glass cell. An Hg/HgO electrode equipped with a Luggin capillary and a Pt foil (2 × 2 cm^2^) electrode were used as the reference and counter electrodes, respectively. All of the potentials were reported with reference to the Hg/HgO electrode. The working electrode was the as-prepared Ag/CFP electrode embedded in a platinum clip, with the active area of 1 cm^2^ immersed in the electrolyte for electrochemical measurements. All the measurements were conducted in an O_2_-saturated 0.1 M NaOH solution and maintained at 25 °C. Cyclic voltammetry (CV), open circuit potential (OCP), linear sweep voltammetry (LSV) and rotating disk electrode (RDE) polarization technique were carried out on a Gamery Reference 3000 electrochemical workstation. Before start of each measurement, the working electrode was immersed in the electrolyte for about 2 h till the OCP was relatively steady. The CV curves for different samples were measured from 0.2 to 0.8 V at a scan rate of 10 mV s^−1^ and scanned for 200 times. The LSV curves were measured from 0.2 to −0.8 V with the sweep rate of 2 mV·s^−1^ and scanned for 10 times. For the RDE polarization curves test, a layer of ground Ag/CFP-3 ethanol solution was directly drop-casted onto the surface of glass carbon electrode (5 mm in diameter) and then wrapped with a drop of 0.5 wt.% Nafion solution to form a working electrode. RDE measurements were carried out with varying rotating speeds from 400 to 2200 rpm by using MSRX Speed Control Unit (Pine Instruments Company). Oxygen gas was maintained over the solution at a flow rate of 30 sccm during the test to ensure its continued O_2_-saturation.

Primary Al-air batteries were tested in a home-built electrochemical cell on a LAND battery testing system under atmospheric air without oxygen feed and at room temperature. The synthesized Ag/CFP-1 (1 cm^2^, catalyst loading 1.02 mg), Ag/CFP-2 (1 cm^2^, catalyst loading 3.56 mg) and Ag/CFP-3 (1 cm^2^, catalyst loading 1.89 mg) were used as the air cathodes. A polished Al plate (4 N, 150 mg in weight, 1 cm^2^, Alfa Aesar) was applied as the anode and a 4 M NaOH aqueous solution was utilized as the electrolyte. For comparison, a commercial nano manganese gas diffusion air cathode (1 cm^2^, 350 µm thick, nano manganese catalyst, Quantum Sphere. Inc.) was measured at the same condition.

## Electronic supplementary material

Supplementary information
