# Supplementary material for: In-situ Electrodeposition of Highly Active Silver Catalyst on Carbon Fiber Papers as Binder Free Cathodes for Aluminum-air Battery
Source: Sci Rep. 2017 Jun 13;7:3378. doi: 10.1038/s41598-017-03609-9 (PMC5469864; doi:10.1038/s41598-017-03609-9)
Supplement: Supplementary file 1 — Supplementary information [file 41598_2017_3609_MOESM1_ESM.doc]

***Electronic Supplementary Information (ESI)***

***In-situ* Electrodeposition of Highly Active Silver** **Catalyst on Carbon Fiber Papers as Binder Free Cathodes for** **Aluminum-air Battery**

Qingshui Hong, Huimin Lu*

*School of Materials Science and Engineering,* *Beihang University, Beijing 100191, China*

** To whom correspondence should be addressed:*[*lhm0862002@aliyun.com*](mailto:lhm0862002@aliyun.com)

The primary Al-air battery with Ag/CFP-3 air cathode was underwent 5 cycles under the same discharge conditions by replenishing the Al anode and electrolyte to determine the variation in the specific capacitance and power density of the battery for a long-term run. As shown in Figure S1, both the specific capacitance (changing within ±0.43%) and power density (changing from 107.5 mW cm^-2^ to 111.8 mW cm^-2^) of the battery degraded slightly.

**
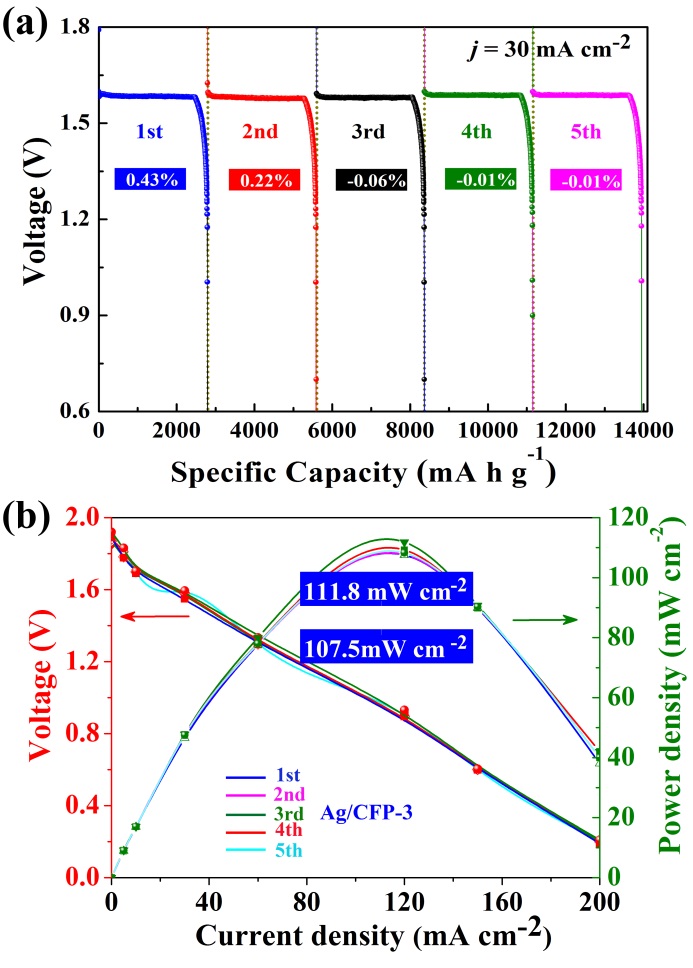
**

**Figure S1.** Performance of the primary Al-air battery fabricated with Ag/CFP air cathode: (a) The variation in the specific capacitance of the battery measured at the current density of 30 mA cm^-2^ with five times replenishing the Al anode and electrolyte; (b) Cell voltage and power density polarization curves of the battery over 5 cycles.

The contact angles of the CFPs before and after oxidation are tested by a home-built method to check the wettability of oxidized CFP in aqueous solution. As shown in Figure S2, a spherical liquid droplet with a smaller contact angle of approximate 90°was formed on the surface of oxidized CFP and the droplet was adsorbed after a while. In contrast, the shape of the drop with a higher contact angle of approximate 160° on the initial CFP was basically unchanged over a long time. The significant change in wettability of oxidized CFP joins the elemental mapping analysis of oxygen element in Figure 4 b3 suggesting the formation of surface oxygen-containing functional groups on CFP, which are able to facilitate the absorption of Ag cation and then form the firm adhesion of Ag catalyst layer.


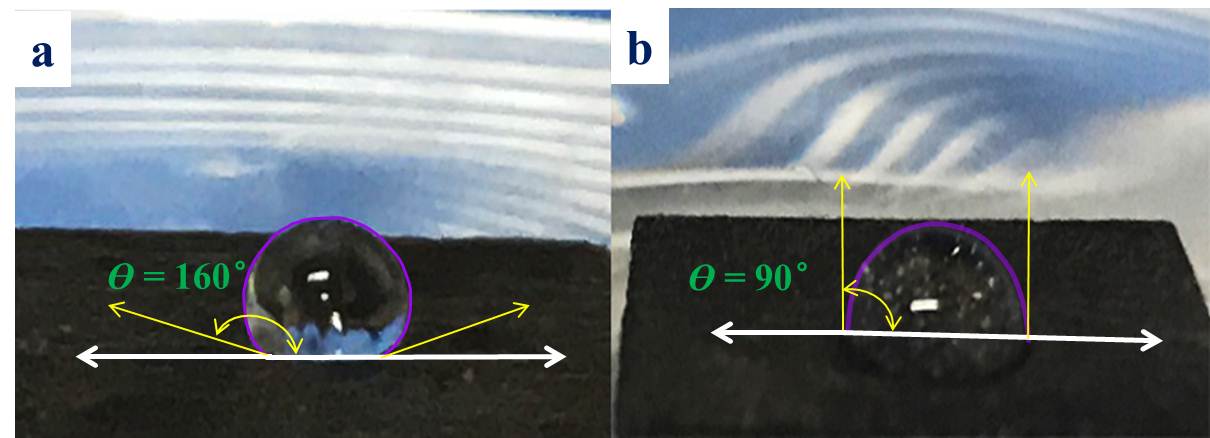


**Figure S2.** The precursor solution contact angles of the CFPs before (a) and after (b) oxidation.
